# Coxsackievirus B3 Directly Induced Th17 Cell Differentiation by Inhibiting Nup98 Expression in Patients with Acute Viral Myocarditis

**DOI:** 10.3389/fcimb.2016.00171

**Published:** 2016-12-09

**Authors:** Qi Long, Yu-Hua Liao, Yu Xie, Wei Liang, Xiang Cheng, Jing Yuan, Miao Yu

**Affiliations:** Laboratory of Cardiovascular Immunology, Institute of Cardiology, Union Hospital, Tongji Medical College, Huazhong University of Science and TechnologyWuhan, China

**Keywords:** viral myocarditis, coxsackievirus B3, Nup98, Th17 cells, coxsackie-adenovirus receptor

## Abstract

Th17 cells play a key role in the progression of coxsackievirus B3 (CVB3)-induced acute viral myocarditis (AVMC). However, the direct effect of virus on Th17 cell differentiation is still unknown. Recently, nucleoporin (Nup) 98 has been proved to be associated with lymphocyte differentiation. Therefore, we investigated whether Nup98 mediated Th17 cell differentiation in AVMC. In our study, patients with AVMC and healthy controls were recruited. The results showed that CVB3 could enter into the CD4^+^ T cells in AVMC patients and healthy controls. After transfecting purified CD4^+^ T cells with CVB3 *in vitro*, the Th17 cell frequency, IL-17 secretion, and RORγT synthesis were increased while the Nup98 levels were decreased. Furthermore, down-regulating Nup98 expression by siRNA-Nup98 in CD4^+^ T cells resulted in the elevated Th17 cell frequency and IL-17 secretion, along with enhanced levels of RORγT, dissociative p300/CBP, and acetylated Stat3. Up-regulation of Nup98 expression by pcDNA3.1-Nup98 showed the opposite effects. Our results suggested that CVB3 directly induced CD4^+^ T cell differentiation into Th17 cells by inhibiting Nup98 expression, representing a therapeutic target in AVMC.

## Introduction

Acute viral myocarditis (AVMC) is triggered by viral infection and is characterized by myocardial inflammation, which progresses to chronic dilated cardiomyopathy (DCM) and heart failure (Dennert et al., 2008). Frequency of myocarditis has been reported to range from a low of 4–5% in young men dying of trauma to a high of 16–21% in children succumbing to sudden death (Gauntt and Huber, 2003). Coxsackievirus B3 (CVB3) is the most commonly identified cause of myocarditis and has been linked to the ensuing development of dilated cardiomyopathy (Esfandiarei and McManus, 2008; Andréoletti et al., 2009; Fairweather et al., 2012). CD4^+^ Th17 cells promote the development of AVMC by facilitating viral replication, inflammation and autoantibody production (Yuan et al., 2010a,b). Previous studies revealed that viral infection induced the activation of innate immune cells such as macrophages and dendritic cells (DCs), which indirectly contribute to Th17 cell differentiation by secreting inflammatory cytokines and generating co-stimulatary signals (Fairweather et al., 2005; Huang and Yang, 2009; Yajima and Knowlton, 2009; Rose, 2011). However, the direct effect of virus on Th17 cell differentiation is still unknown.

Nuclear pore complexes (NPCs) are multi-protein aqueous channels connecting the nucleus and cytoplasm, mediating cell differentiation (Raices and D'Angelo, 2012). NPCs consist of nearly 30 different proteins called nucleoporins (Nups) (Kalverda and Fornerod, 2007). Among the Nups, Nup98 is associated with mRNA export pathway induced by viruses (Enninga et al., 2002). Enninga et al. have shown that vesicular stomatitis virus (VSV) matrix (M) contributes to Nup98 expression and directly induces antiviral cytokine IFN-γ generation by inhibiting messenger RNA (mRNA) nuclear export (Enninga et al., 2002). Therefore, we investigated the role of Nup98 in virus-induced differentiation of Th17 cells in AVMC patients.

## Materials and methods

### Patients

A total of 21 patients at the Union Hospital, Huazhong University of Science and Technology were diagnosed with AVMC and enrolled in our study from June 2014 to January 2015. All of them had developed symptoms associated with heart within 3 weeks after viral infection such as upper respiratory tract infection and diarrhea. The diagnosis was established according to the “Reference standards for diagnosis of acute viral myocarditis in adults” recommended by Committee of Task Force on Myocarditis and Cardiomyopathy of Chinese Journal of Cardiology Editor (1999), which was consistent with the recommendations of the 6th edition of *Braunwald's Heart Disease* (Wynne and Braunwald, 2001). In addition, all the patients diagnosed with AVMC exhibited a mild increase in creatine kinase (CK) MB or cardiac troponin I (cTNI) at the time of enrollment. The plasma of all the AVMC patients tested CVB3-IgM positive (Patients with CVB5-IgM positive, Cytomegalovirus-IgM positive and Parvovirus B19-IgM positive were all excluded from our study). Patients with other acute or chronic diseases were excluded and no patient was treated with nonsteroidal anti-inflammatory drugs or immunosuppressors. Furthermore, 23 volunteers were recruited as controls in the study. This study was first conducted in accordance with the tenets of the Declaration of Helsinki and its amendments and was subsequently approved by The Ethics Committee of Tongji Medical College, Huazhong University of Science and Technology, China (IORG No: IORG0003571). Each recruit provided signed informed consent.

### Blood samples

Blood samples were obtained from all the patients and healthy controls in the recumbent position under fasting state the next morning of hospitalization. The blood samples were stored in vacutainer tubes containing 3.2% sodium citrate. Each blood sample was centrifuged at 2000 rpm for 15 min. The plasma was collected for cytokine measurement. The blood cells were layered over Ficoll-Hypaque density gradient solution to separate peripheral blood mononuclear cells (PBMCs) for flow cytomentry, magnetic cell sorting, real time-polymerase chain reaction (RT-PCR) and Western blot.

### ELISA

The plasma levels of IL-17 were measured using the enzyme-linked immunosorbent assay (ELISA) kit (ebioscience), according to the manufacturer's instructions. The ELISA kit showed a sensitivity of 1.6 pg/mL. All the samples were analyzed in triplicate.

### Immunoturbidimetric assay

Plasma hsCRP (hypersensitive C reactive protein) were measured by Beckman AU 5800 using immunoturbidimetric assay (Beckman Coulter Inc) according to the manufacturer's instructions. The sensitivity of hsCRP was 0.11 mg/L (Karaca et al., 2016).

### Isolation of human CD4^+^ T cells

The peripheral blood cells obtained from healthy controls and AVMC patients were layered over Ficoll-Hypaque density gradient solution (Sigma) in order to obtain mononuclear cells. The CD4^+^ T cells were purified by negative selection using human CD4^+^ T cell isolation kit (Miltenyi Biotech) according to the manufacturer's protocol. Briefly, PBMCs were incubated with CD4^+^ T cell biotin-antibody cocktail (10 μl/10^7^cells) for 5 min, followed by anti-biotin microbeads (40 μl/10^7^cells) for 10 min at 4°C. After washing with MACS buffer, the re-suspended cells were loaded on an LS column (Miltenyi Biotech) to obtain the purified CD4^+^ T cells (purity > 95%).

### CVB3-infected CD4^+^ T cells

The CD4^+^ T cells from healthy controls were cultured at 5 × 10^5^ cells/mL for 12 h at 37°C in six-well plates (Costar). For experimental infections, cells were washed once with serum-free 1640 medium (Hyclone). The 0.1 mL 1640 medium containing CVB3 (CCTCC, GDV115, 5 × 10^5^ plaque forming unit (PFU)/mL) was added to CVB3 group, and 0.1 mL 1640 medium without virus was added to the mock group. This system was cultured for 2 h in 1 mL serum-free 1640 medium. After washing, cells were cultured with 1640 medium containing 5% FBS, 5 μg/mL of anti-CD3 (ebioscience), 2 μg/mL soluble anti-CD28 (eBioscience), 10 μg/mL anti-IL-4 (ebioscience), and 10 μg/mL anti-IFN-γ (ebioscience) for 5 days at room temperature. The cells and culture supernatants were harvested for further analysis. The virus experiment was performed according to the general requirements for laboratory biosafety (GB 19489-2008) in China.

### Plaque-forming assay

10^5^ CD4^+^ T cells were homogenized in 1 mL 1640 medium. The virus was released from the cells following freeze-thaw cycles and the supernatant was obtained. The HeLa cell monolayers (70% confluency) were incubated with supernatants of infected CD4^+^ T cells for 2 h at 37°C and 5% CO_2_, in 24-well plates. After washing with PBS, plates were covered with a 3 mL mix of 0.3% agar, 1640, and 5% FBS. After 72 h of cultivation, the monolayers were fixed and stained in neutral red, and the plaques were counted. Viral titers were determined using standard plaque formation assay.

### Transfection

After isolation, the purified CD4^+^ T cells from AVMC patients were transferred into 1640 medium with 10% FBS at a density of 3 × 10^6^ cells /mL in a 12-well culture plate (Corning) and cultured at 37°C/5% CO_2_. They were transfected with 200 nM siRNA-Nup98 (IBS company, sense: GGAUGACCGAGAAGAAAUAGA, antisense: UAUUUCUUCUCGGUCAUCCUG) or 4 μg pcDNA3.1-Nup98 plasmid (IBS company) using the Amaxa human T-cell nucleofector kit (Lonza Cologne AG) via V24 program according to the manufacturer's instructions. 4 μg pmaxGFP® Vector was transfected into 3 × 10^6^ CD4^+^ T cells by necleofection. The transfection efficiency was evaluated by flowcytometry. After transfected 12 h, green fluorescent protein (GFP) expression was checked to show the efficiency. The nonsilencing control (NC) siRNA (IBS company, sense: UUCUCCGAACGUGUCACGUTT, antisense: ACGUGACACGUUCG GAGAATT) or empty pcDNA3.1 (IBS company) was transfected into CD4^+^ T cells as control. Cells were then stimulated with 5 × 10^4^ PFU/mL CVB3, 5 μg/mL of anti-CD3 (ebioscience), 2 μg/mL soluble anti-CD28 (eBioscience), 10 μg/mL anti-IL-4 antibody (ebioscience), and 10 μg/mL anti-IFN-γ antibody (ebioscience). After 72 h of incubation, cells and supernatants were collected, respectively, for further study.

### Flow cytometry

The PBMCs or CD4^+^ T cells were re-suspended to approximately 2 × 10^6^ cells in 100 μL ice-cold PBS. Fluorescein isothiocyanate (FITC)-labeled anti-human CD4 antibody (Biolegend) was added to each tube along with Phycoerythrin-Cy7 (PE-CY7) anti-human CD45RO antibody (BD biosciences). The cells were incubated for 30 min at 4°C in the dark. After washing with PBS, these cells were stimulated with 1 μg/mL ionomycin, 20 ng/mL phorbol myristate acetate (PMA), and 2 μmol/L monensin (all from eBioscience) for 5 h under a 5% CO_2_ /37°C environment in a 24-hole culture plate (Costar). After 5 h, cells were harvested and washed with PBS. They were fixed and permeabilized by FACS™ Perm 2 (BD Bioscience) according to the manufacturer's instructions. For intracellular staining, the cells were stained with phycoerythrin (PE)-labeled anti-human IL-17 antibody (BD biosciences) for 30 min. After washing with PBS, these cells were re-suspended with 200 μL PBS in tube, respectively. After adding 10 μl CountBright™ Absolute Counting Beads (Thermo Fisher) into each tube, samples were measured by FACS calibur flow cytometry (BD Biosciences).

### Western blot

The total proteins in PBMCs or CD4^+^ T cells were extracted using the Total Protein Extraction Kit (Pierce/Thermo Scientific). BCA Protein Assay Kit (Pierce) was used to determine protein concentrations. Samples containing 60 μg protein were separated on 10% SDS-PAGE or 8% SDS-PAGES and electrotransferred onto nitrocellulose membranes. The membranes were blocked for 2 h in TBST containing 5% skim milk and then incubated with primary antibodies at 4°C overnight. The primary antibodies Nup98 (1:800, Abcam), RORγT (1:500, Abcam), p300/CBP (1:500, Sigma), Acetyl-Stat3 (1/500, Thermo scientific), Stat3 (1:1000, Cell Signaling Technology), CAR (1:500, Santa Cruz), DAF (1:1000, Abcam), and β-actin antibodies (1:1000, Millipore) were added, respectively. After washing, the membranes were incubated with HRP-conjugated secondary antibodies (1:3000, Jackson ImmunoResearch) at 37°C for 2 h. The target bands were finally washed and developed with ECL reagent or super ECL reagent (Thermo Scientific), and then captured using Image Lab (Bio-Rad). Densitometric methods were used to analyze the images.

### Real-time PCR

The total RNA was extracted from the cells using the TRIzol reagent (Takara) following the protocol and then reverse transcribed to cDNA. The sequences of primer pairs were as follows: Nup98 (forward) 5′-CTCCACCACTAATTCAGGCTTT-3′ and Nup98 (reverse) 5′-GAGGCTGGTAGTCTGCTGATT-3′; RORγT (forward) 5′-CAAAGCAGGAGCAATGGAAGTG-3′ and RORγT (reverse) 5′-GGAGTGGGAGAAGTCAAAGATG-3′; IL-17 (forward) 5′-CCTCAGACTACCTCAACCGTTC-3′ and IL-17 (reverse) 5′-TTCATGTGGTGGTCCAGCTTTC-3′; β-actin (forward) 5′-AAGGCCAACCGTGAAAAGAT-3′and β-actin (reverse) 5′-GTGGTACGACCAGAGGCATAC-3′; CVB (forward)5′-CGGTACCTTTGTGCGCCTGT-3′ and CVB (reverse) 5′-CAGGCCGCCAACGCAGCC-3′. After an initial denaturation step at 94°C for 3 min, 40 cycles were carried out, each of which consisted of the following three steps: denaturation at 94°C for 30 s, annealing at 58°C for 30 s, and extension at 72°C for 30 s. All the reactions were conducted at least in duplicate for each sample. The gene expression was analyzed using the real-time fluorescent quantitative PCR system (Bio-rad). The level of gene expression was calculated by the 2^−ΔΔCt^.

### Statistical analysis

Data were represented as the mean ± SEM. Statistical analysis was conducted using a two-tailed Student's *t*-test for two groups and one-way ANOVA for more than two groups. Chi-square test was performed for the categoric variables of clinical characteristics. Bivariate correlation analysis was used as a test of correlation between two variables. *P* < 0.05 was considered statistically significant.

## Results

### Viral detection in CD4^+^ T cells of AVMC patients

The clinical data of AVMC patients and healthy volunteers are displayed in Table 1. No significant differences were found in age and sex. Compared with healthy controls, the levels of plasma hsCRP (*P* < 0.001) and IL-17 (*P* < 0.001) were higher in patients with AVMC.

**Table 1:** **Clinical Characteristics of AVMC Patients and Healthy controls**.

**Characteristics**	**Healthy controls (*n* = 23)**	**AVMC patients (*n* = 21)**
Age (year)	36 ± 5	34 ± 17
Gender (Male:Female)	9:14	8:13
Hospital stays (day)	0(0)	9 ± 4
**MAJORORGAN INVOLVEMENT**		
Pulmonary. No (%)	0(0)	5(23.8)
Kidney. No (%)	0(0)	3(19)
Liver. No (%)	0(0)	0(0)
**NYHA CLASSIFICATION**		
I. No (%)	0(0)	7(33.3)
II. No (%)	0(0)	8(38.1)
III. No (%)	0(0)	0(0)
IV. No (%)	0(0)	0(0)
**MEDICATIONS**		
ACEI/ARBs. No (%)	0(0)	10(47.6)
β-blockers. No (%)	0(0)	18(85.7)
Calciumchannelblockers. No (%)	0(0)	7(33.3)
Nitrates. No (%)	0(0)	3(19.0)
Aldactone. No (%)	0(0)	19(90.47)
CoQ_10_. No (%)	0(0)	21(100)
Diuretica. No (%)	0(0)	7(33.3)
hsCRP (mg/L)	0.49 ± 0.15	5.21 ± 1.68
IL-17 (ng/ml)	26.39 ± 4.88	92.2 ± 8.99

**Figure 1:**
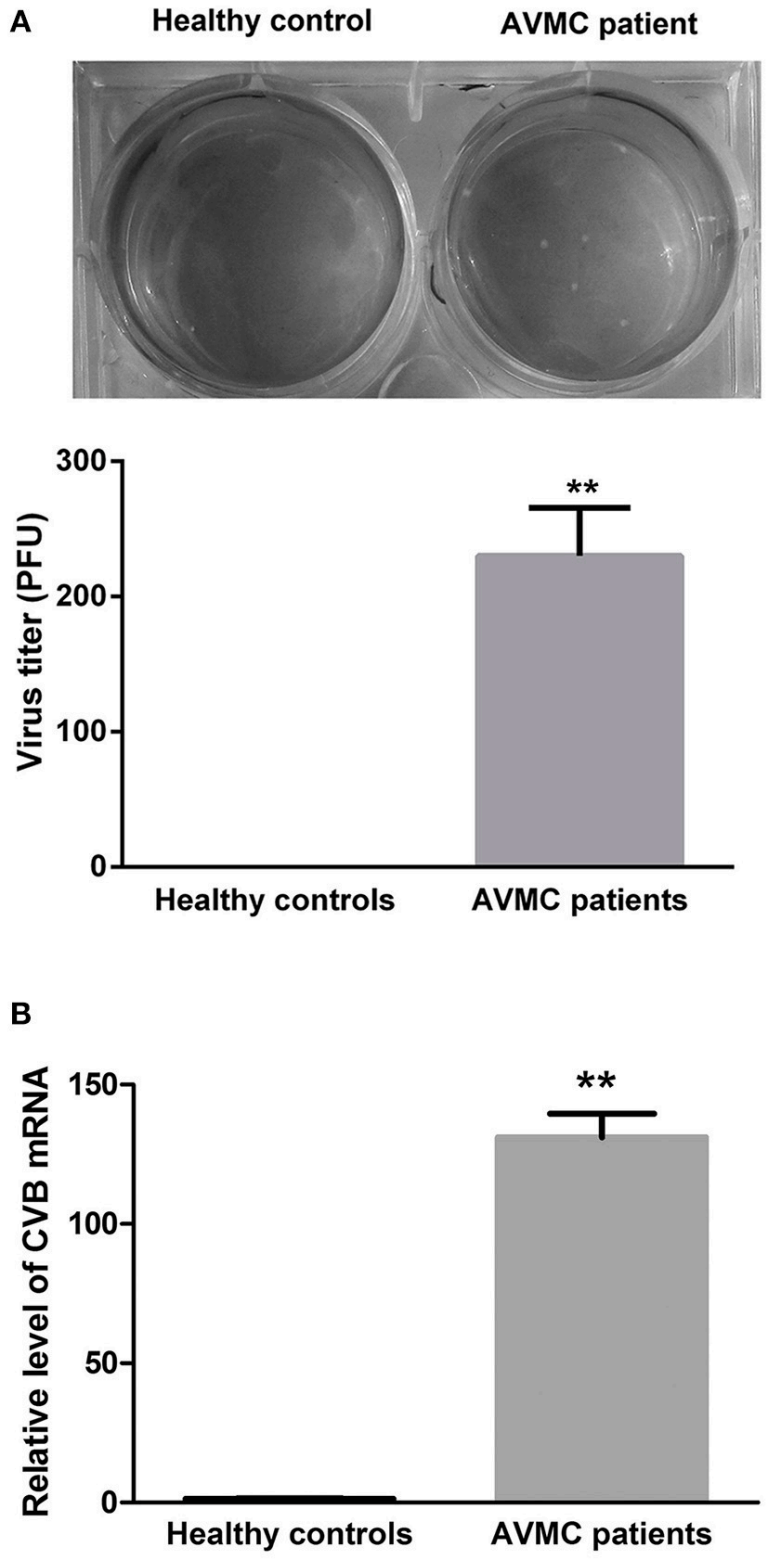
**The virus detection in CD4^**+**^ T cell from AVMC patients. (A)** The CVB3 titers of purified CD4^+^ T cells isolated from peripheral blood in healthy controls and AVMC patients. **(B)** The levels of CVB3 mRNA in CD4^+^ T cell isolated from peripheral blood were higher in AVMC patients than those in healthy controls (*p* < 0.001). Values are means ± SEM. ^**^P < 0.01 vs. Healthy controls.

Purified CD4^+^ T cells isolated from the peripheral blood in AVMC patient and healthy volunteer were collected for viral detection. The data indicated that the average virus titer was 230 PFU/ml (10^5^ cells homogenized in 1 mL 1640 medium) in AVMC patients whereas no virus was detected in healthy volunteers (*P* < 0.001, Figure 1A). In addition, levels of CVB mRNA in CD4^+^ T cell were higher in AVMC patients than those in healthy controls (*P* < 0.001, Figure 1B). These data revealed that virus could be detected in peripheral CD4^+^ T cells of AVMC patients.

### Th17 cell frequencies and Nup98 expression in AVMC patients

**Figure 2:**
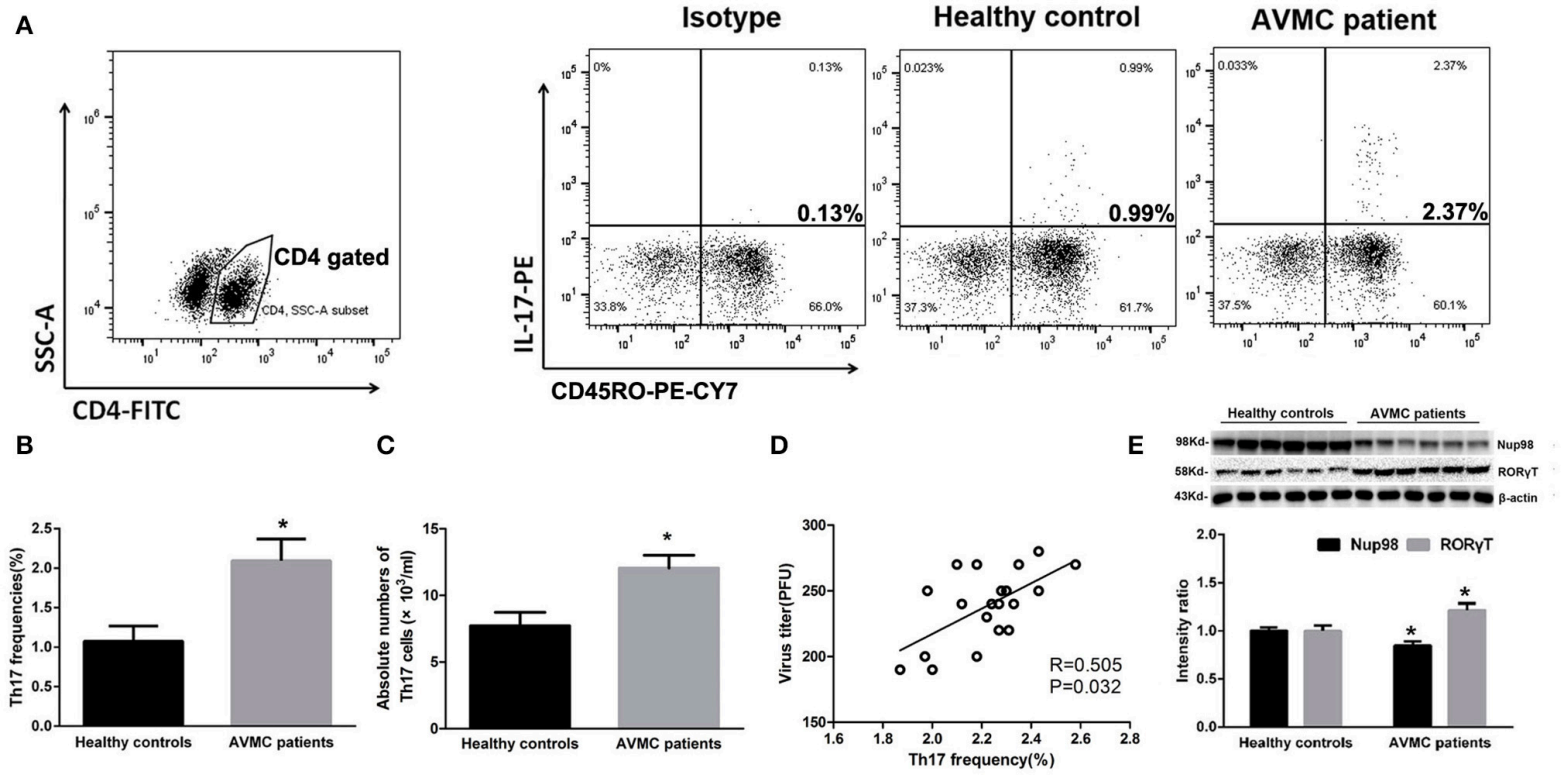
**Th17 cell frequencies and Nup98 expression in AVMC patients. (A)** The CD4^+^ T cells were gated from PBMCs for further analysis. The representative pictures for CD4^+^ CD45RO^+^ Th17 cell frequencies in healthy controls and AVMC patients. **(B)** The results of statistical analysis for Th17 cell frequencies by flow cytometry between healthy controls and AVMC patients were shown in histogram. **(C)** The absolute numbers of Th17 cells (per ml blood) of healthy controls and AVMC patients were shown in the histogram. **(D)** The correlation analysis between Th17 frequencies and virus titers in peripheral CD4^+^ T cells of AVMC patients. **(E)** The representative pictures for Nup98 and RORγT protein expression with western blot analysis.Intensity of bands were normalized to β-actin and shown in histogram. Values are means ± SEM. ^*^P < 0.05 vs. Healthy controls.

To clarify the relationship between Th17 cell frequencies and Nup98 expression, we performed flow cytometry and western blot. Human naive and effector T cells can be identified by CD4^+^CD45RO^−^ T cells and CD4^+^CD45RO^+^ T cells (Teteloshvili et al., 2015). As shown in Figure 2A, after gating CD4 positive cells, we costained IL-17 with CD45RO and found that the frequencies of CD45RO^+^ T cells were about 60%. Furthermore, IL-17 was mostly secreted by CD4^+^ CD45RO^+^ effector T cells rather than CD4^+^ CD45RO^−^ T cells. In PBMCs, the frequencies of CD4^+^CD45RO^+^IL-17^+^ effector (Th17) cells (*P* = 0.014) were increased in AVMC patients compared with healthy volunteers (Figures 2A,B). By using counting beads, we found that the absolute number of Th17 cells was also higher in AVMC patients than that of healthy volunteers (*P* = 0.012, Figure 2C). And the Th17 frequencies were positively correlated with CVB3 virus titers in peripheral CD4^+^T cells of AVMC patients (*R* = 0.505, *P* = 0.032, Figure 2D).

The Nup98 expression in purified CD4^+^ T cells was lower (*P* = 0.018) in AVMC than in healthy volunteers (Figure 2E). However, RORγT, the primary transcription factor of Th17 cell demonstrated the contrary results (*P* = 0.033, Figure 2E). These data indicated that Nup98 expressions were associated with Th17 cell frequencies in AVMC patients.

### Direct effects of CVB3 on Th17 cell differentiation

**Figure 3:**
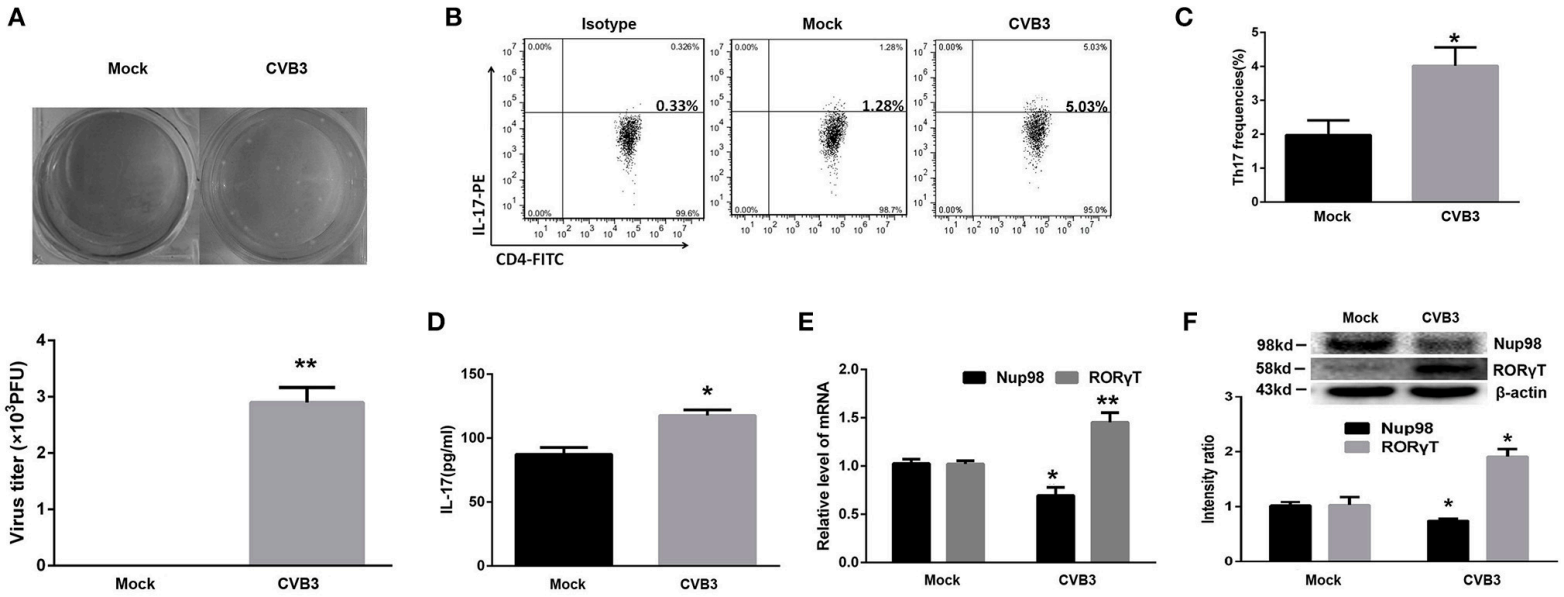
**Direct effects of CVB3 on Th17 cell differentiation. (A)** Upper: Representative pictures of plaque assay of mock-infected and CVB3-infected CD4^+^ T cells from peripheral blood in healthy controls. Lower: The results of statistical analysis for plaque assays in mock and CVB3 groups. **(B)** Representative pictures for Th17 cell frequencies in mock and CVB3 groups by flow cytometry. **(C)** Statistical analysis for Th17 frequencies was shown in histogram. **(D)** The cultural supernatant IL-17 concentration in mock and CVB3 groups was measured by ELISA. **(E)** The mRNA levels of Nup98 and RORγT in CD4^+^ T cells. **(F)** The protein expression of Nup98 and RORγT in CD4^+^ T cells. Values are means±SEM. ^*^P < 0.05 vs. mock group; ^**^P < 0.01 vs. mock group.

The purified CD4^+^ T cells isolated from healthy volunteer blood were collected and mock-infected or directly infected with CVB3. After 5 days, the viral titer was detected. The data showed that the average CVB3 titer was 3.1 × 10^3^ PFU/ml (10^5^ cells homogenized in 1 mL 1640 medium) in the CVB3 group while no plaque was formed in the mock group (Figure 3A). The frequencies of Th17 cells in CVB3 group were increased (*P* = 0.021) compared with those in the mock group (Figures 3B,C). The IL-17 levels in the supernatant (*P* = 0.013) showed similar changes (Figure 3D). The mRNA (*P* = 0.009) and protein (*P* = 0.009) levels of RORγT in the CVB3 group were enhanced compared with those in the mock group (Figures 3E,F). However, the Nup98 mRNA (*P* = 0.040) and protein (*P* = 0.030) levels showed the opposite changes (Figures 3E,F). From this, we found that CVB3 could directly induced Th17 cell differentiation, which might be related to the decreased Nup98 expression.

### The expression of CVB3 receptors on CD4^+^ T cells

**Figure 4:**
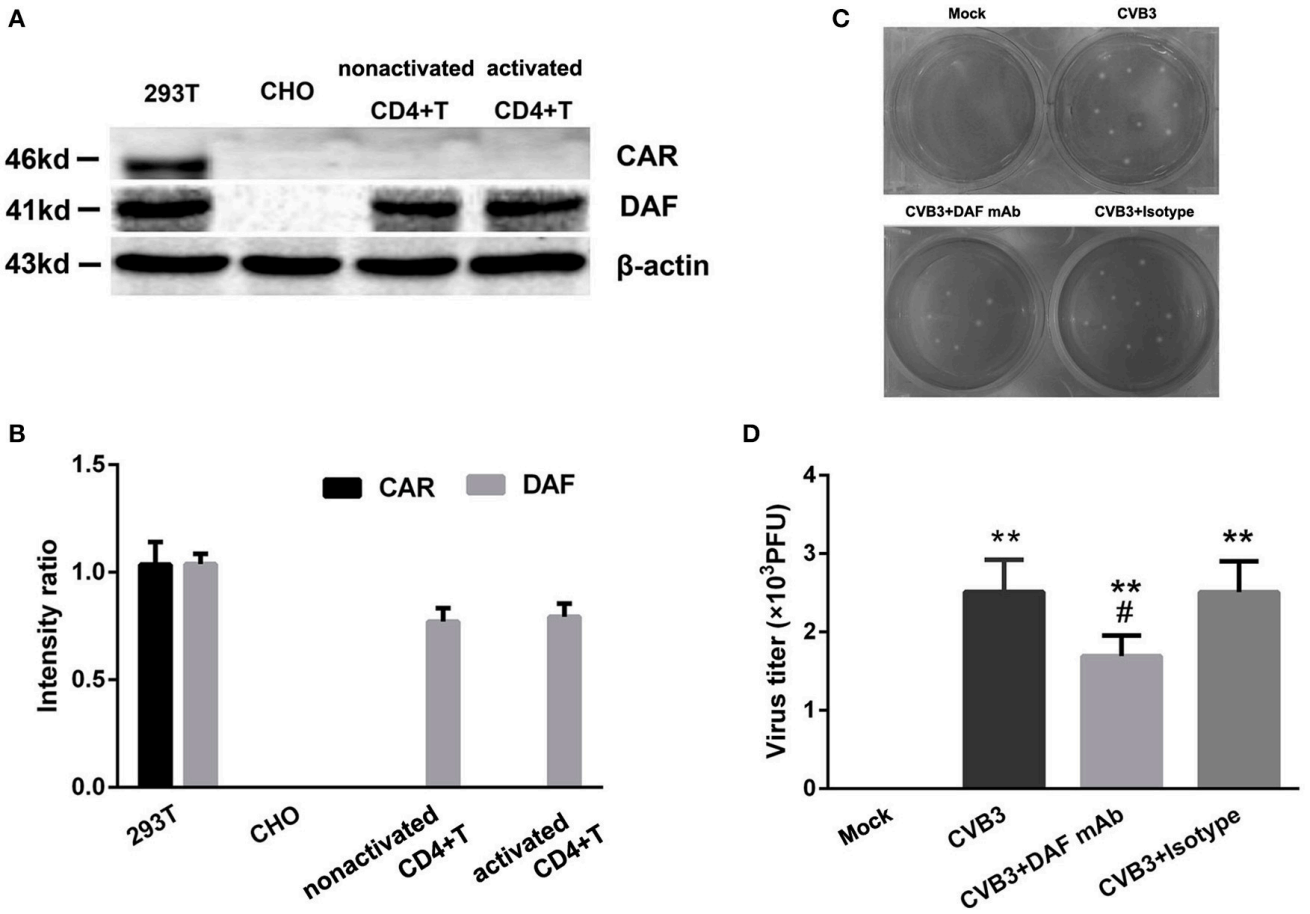
**The expressions of CVB3 receptors on CD4^**+**^ T cells. (A)** The expressions of CAR/DAF on nonactivated and activated CD4^+^ T cells were investigated. 293T and CHO cell lines were used as the CAR/DAF -positive and -negative controls respectively. **(B)** The CAR was not expressed on both nonactivated and activated CD4^+^ T cells (5 μg/ml anti-CD3, 2 μg/ml anti-CD28 stimulated for 3 days). The DAF was expressed on both nonactivated and activated CD4^+^ T cells, but the expressions of DAF between nonactivated and activated CD4^+^ T cells showed no difference. **(C)** The representative pictures of plaque assay for purified CD4^+^ T cells in mock, CVB3, CVB3+DAF mAb, CVB3+Isotype groups (all CD4^+^ T cells were isolated from peripheral blood in healthy controls). **(D)** The results of statistical analysis for CVB3 titers in these four groups. Values are means ± SEM. ^**^P < 0.01 vs. Mock group; ^#^*P* < 0.05 vs. CVB3 and CVB3+Isotype groups.

The expression of the primary receptor coxsackie-adenovirus receptor (CAR) of CVB3 on CD4^+^ T cells was determined. The cell lines 293T and Chinese hamster ovary (CHO) were used as CAR positive and negative controls, respectively (Candolfi et al., 2006). However, we found that neither nonactivated CD4^+^ T cells nor activated CD4^+^ T cells expressed CAR (Figures 4A,B). Decay-accelerating factor (DAF) is a binding receptor for CVB3. The 293T cells and Chinese hamster ovary (CHO) cells were also used as DAF-positive and -negative controls for DAF, respectively (Bétis et al., 2003). The DAF could be detected on CD4^+^ T cells. However, the expression of DAF showed no differences (*P* = 0.810) between nonactivated CD4^+^ T cells and activated CD4^+^ T cells (Figures 4A,B). To further investigate the role of DAF in CVB3 infection, the 10 ug/ml anti-DAF monoclonal antibodies (mAb, R&D), 10 ug/ml isotype control and 10 ul saline was added in the culture system respectively. The data showed that the CVB3 titer in CD4^+^ T cells treated with anti-DAF mAb was significantly lower (*P* = 0.043) than those treated with saline and isotype controls (Figures 4C,D). These data suggested that the entry of CVB3 into human CD4^+^ T cells might be mediated by DAF receptors rather than CAR.

### Effect of Nup98 on Th17 cell differentiation in AVMC

**Figure 5:**
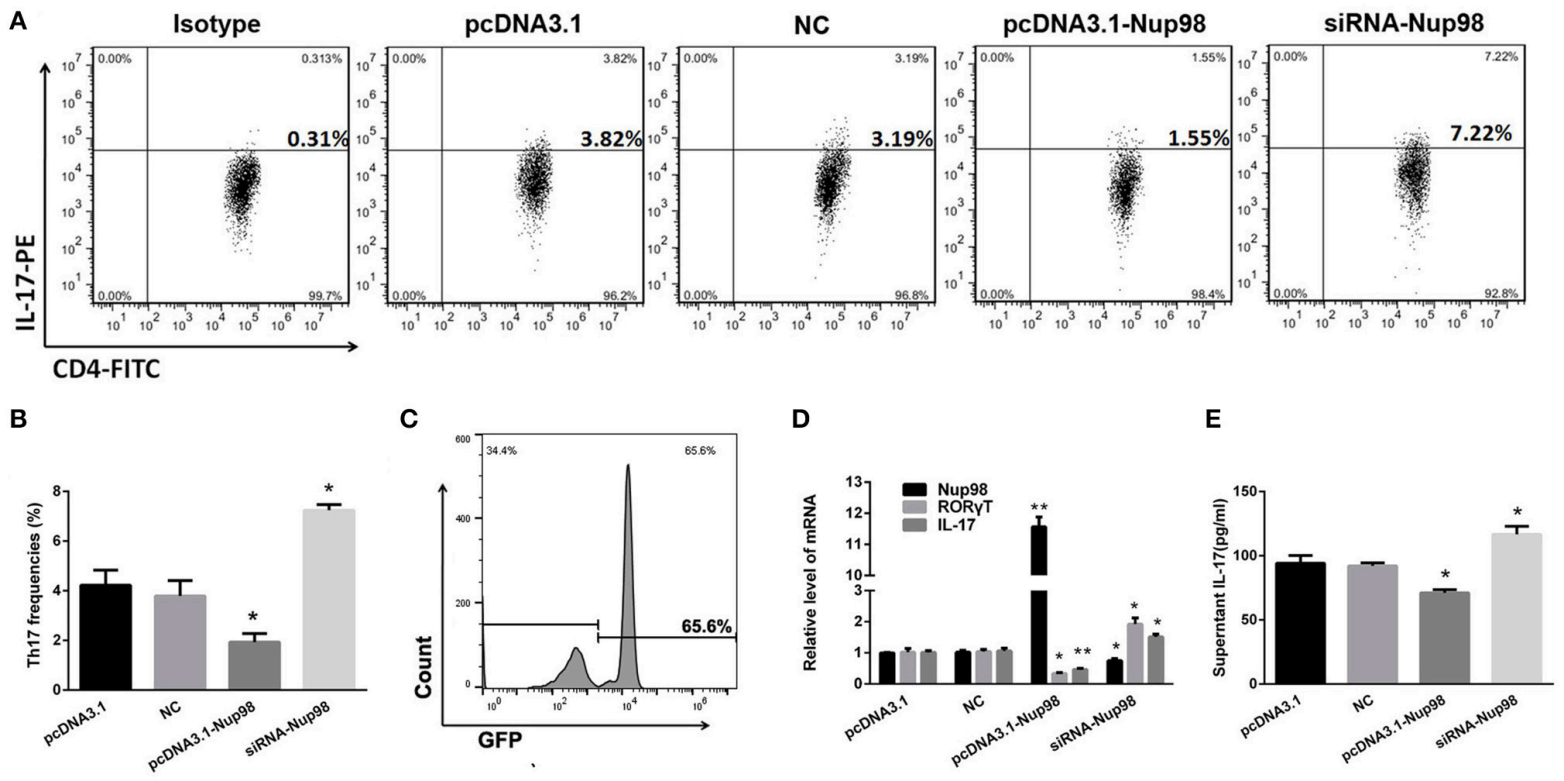
**Effects of Nup98 on Th17 cell differentiation in AVMC**. CVB3 infected CD4^+^ T cells from peripheral blood in AVMC patients were transfected with Nup98 siRNA or Nup98 cDNA by the Human T Cell Nucleofector Kit and cultured with anti-CD3, anti-CD28, anti-IL-4 and anti-IFN-γ for 72 h. **(A)** Representative pictures of Th17 cell frequencies of CD4^+^ T cell in pcDNA3.1, NC, pcDNA3.1-Nup98, and siRNA-nup98 groups were listed. **(B)** The statistical analysis for Th17 frequencies was shown in histogram. **(C)** The GFP-transfection efficiency was detected by flow cytometry and a typical FACS picture was shown. **(D)** The mRNA levels of Nup98, RORγT and IL-17 in CD4^+^ T cells of four groups. **(E)** The cultural supernatant IL-17 concentration was measured by ELISA. Data are shown as mean ± SEM. ^*^P < 0.05, ^**^P < 0.01 vs. pcDNA3.1 and NC groups. NC: Nonsilencing siRNA sense.

To further clarify the role of Nup98 in Th17 cell differentiation in AVMC, the purified CD4^+^ T cells from AVMC patients were transfected with empty pcDNA3.1, pcDNA3.1-Nup98, NCsiRNA and siRNA-Nup98. The frequencies of Th17 cells were higher (*P* = 0.043) in the Nup98-siRNA group but lower (*P* = 0.021) in the pcDNA3.1-Nup98 group compared with those in pcDNA3.1 and NC groups (Figures 5A,B). Compared with pcDNA3.1 and NC groups, the levels of Nup98 mRNA in pcDNA3.1-Nup98 group were enhanced (*P* = 0.010), while the Nup98 mRNA in siRNA-Nup98 was reduced (*P* = 0.040, Figure 5D). The levels of IL-17 in the supernatant, IL-17 mRNA and RORγT mRNA in CD4^+^ T cells were altered in accordance with Th17 cells (all *P* < 0.05, Figures 5D,E). The differences between NC and empty pcDNA3.1 groups were not statistically significant (Figure 5). As a result, Th17 cell differentiation could be induced by down-regulating Nup98 expression after CVB3 infection.

### Nup98 pathway in Th17 differentiation

**Figure 6:**
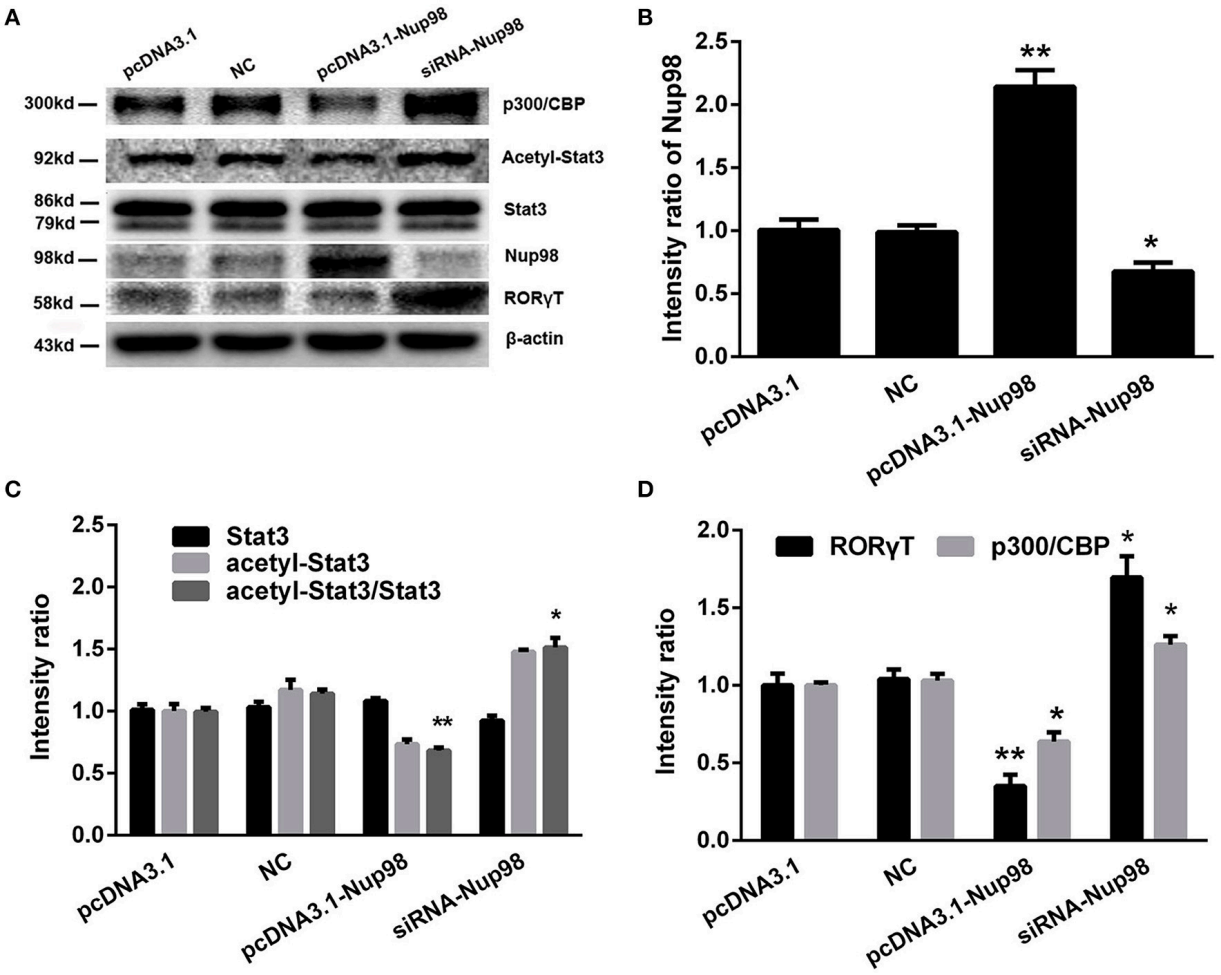
**Nup98 pathway in Th17 differentiation. (A)** Representative pictures for the expressions of p300/CBP, acetyl-Stat3, Stat3, and RORγT in CD4^+^ T cells from peripheral blood in AVMC patients with different interventions. Intensity of each band was measured and normalized to the expression of β-actin. **(B)** Results of the statistical analysis for the level of Nup98. **(C)** Results of the statistical analysis for the level of acetyl-Stat3. **(D)** Results of the statistical analysis for the level of RORγT and p300/CBP. Values represent means ± SEM. ^*^P < 0.05, ^**^P < 0.01 vs. pcDNA3.1 and NC groups. NC: Nonsilencing siRNA sense.

Compared with pcDNA3.1 and NC groups, the levels of dissociative p300/CBP, acetylated-Stat3, and RORγT were decreased along with the increased Nup98 expression in pcDNA3.1-Nup98 group (all *P* < 0.05, Figure 6). Furthermore, down-regulation of Nup98 expression resulted in opposite changes. The differences between NC siRNA and pcDNA3.1 groups were not significant (Figure 6). Thus, CVB3 directly induced Th17 cell differentiation by promoting acetyl-Stat3-mediated RORγT synthesis.

## Discussion

Early studies showed that CVB3 VP1 directly stimulated T cell proliferation *in vitro* (Huber et al., 1993). Kemball et al. further demonstrated that CVB3 infection directly induced the differentiation and generation of virus-specific memory CD4^+^ T cells (Kemball et al., 2009). Nevertheless, our understanding of the direct effects of CVB3 on CD4^+^ T cells remains unclear. In this study, we found that CVB3 was present in CD4^+^ T cells of AVMC patients. Moreover, CVB3 entered CD4^+^ T cells and directly induced CD4^+^ T cell differentiation into Th17 cells.

The CAR allows internalization of the CVB3 after attachment and is the common pathway mediating viral entry into the cell (Liu and Mason, 2001). However, we found that CAR was undetectable in nonactivated and activated CD4^+^ T cells. DAF, a co-receptor of CAR, is expressed on CD4^+^ T cells and facilitates CVB3 internalization by increasing the binding efficiency of CVB3 on the DAF-CAR complex. Although Patel et al. considered that the interaction between CVB3 and DAF alone was insufficient for CVB3 infection (Patel et al., 2009), Pan et al. indicated that CVB3 infection of human intestinal epithelial cells without CAR expression was dependent on DAF (Pan et al., 2015). Furthermore, Martino et al. showed that anti-DAF mAb blocked coxsackie virus infection of susceptible HeLa cells by reducing viral adhesion and internalization (Martino et al., 1998). In this study, we also found that anti-DAF mAb attenuated CVB3 infection in CD4^+^ T cells. These data suggested that the entry of CVB3 into human CD4^+^ T cells might be mediated by DAF receptors, not by CAR. In addition, Sims et al. showed that exosomes significantly enhanced adenoviral entry in CAR-deficient cells in a receptor-independent fashion (Sims et al., 2014), which provide another pathway for CVB3 entry into CD4^+^ T cells. The precise mechanisms are under further investigation.

Nup98 is an important nucleoporin mediating nuclear translocation of mRNAs in viral infection (Enninga et al., 2002). In the course of investigation of the direct effects of CVB3 on Th17 cells differentiation, we found that Nup98 expression was decreased while Th17 cell frequencies and IL-17 levels were increased in AVMC patients. In addition, the CVB3 virus titers in peripheral CD4^+^ T cells were positively correlated with Th17 frequencies in AVMC patients. After CVB3 infection *in vitro*, CVB3 infection also reduced the Nup98 expression but enhanced Th17 cell differentiation and IL-17 secretion. Thus, we deduced that Nup98 plays a role in CVB3-mediated Th17 cell differentiation.

To clarify the direct effect of Nup98 on Th17 cell differentiation, we transfected the pcDNA3.1-Nup98 or siRNA-Nup98 into CD4^+^ T cell to overexpress or silence Nup98. Down-regulation of Nup98 expression facilitated Th17 cell differentiation and IL-17 secretion, and up-regulation of Nup98 expression inhibited Th17 cell differentiation and IL-17 secretion. The findings suggest that CVB3 infection directly induced Th17 cell differentiation by down- regulation of Nup98 in CD4^+^ T cells in AVMC.

RORγT is the key transcription factor in Th17 cell differentiation (Ivanov et al., 2006). Activation of Stat3 leads to increased expression of orphan nuclear receptors RORγT for Th17 cell (Chaudhry et al., 2009). Nup98 interacted with p300/CREB binding protein (CBP) via the region designated as the FG (Phe–Gly) repeat, which is associated with RORγT synthesis (Goodman and Smolik, 2000; Ciofani et al., 2012). Furthermore, the dissociated CBP activated Stat3 via acetylation. The acetylated-Stat3 is transported into nucleus to bind with the promoter region of RORγT, triggering Th17 cell differentiation (Wang et al., 2005). Thus, we speculated that Nup98/p300/ acetylated-Stat3 pathway might be involved in CVB3-induced Th17 cell differentiation. After identifying these signaling molecules, we found that the siRNA-Nup98 reduced Nup98 expression, attenuated the binding of Nup98 to CBP, enhanced dissociative CBP levels, and upregulated acetylated-Stat3, which promoted RORγT expression. It indicates that CVB3 directly induced Th17 cell differentiation by promoting acetyl-Stat3-mediated RORγT synthesis.

In addition, studies suggested that hypoxia-inducible factor (HIF)-1 recruited p300 and RORγt transcription complex to IL-17 promoter and facilitated IL-17 production (Dang et al., 2011). Thus, we speculated that Nup98 competitively bound to p300 and inhibited the formation of p300-HIF-RORγt complex, which reduced Th17 cell differentiation and IL-17 secretion. However, elucidation of the mechanism underlying these changes needs additional investigations.

As a limitation of this study, CD4^+^ T cells were all isolated from human peripheral blood. Further study about CD4^+^ T cells isolated from hearts of myocarditis patients by endomyocardial biopsy will be more valuable. In addition, there are up to 20 known viruses that may elicit myocarditis (Biesbroek et al., 2015; Tse et al., 2016). However, we only investigated the direct effect of CVB3 on Nup98 expression and CD4^+^ T cell differentiation. The influences of other viruses on CD4^+^ T cell differentiation into Th17 cell needed our further exploration. At the same time, the influences of CVB3 on the other cytokines related to Th17 cell such as IL-6, IL-17F, IL-22, and IL-23 are also under investigation. And the direct effects of CVB3 on Th1 cell need our further exploration.

## Ethics statement

This study was first conducted in accordance with the tenets of the Declaration of Helsinki and its amendments and was subsequently approved by The Ethics Committee of Tongji Medical College, Huazhong University of Science and Technology, China (IORG No: IORG0003571). Each recruit provided signed informed consent.

## Author contributions

MY, JY, and YL conceived and designed the experiments. QL, YX, and WL performed the experiments. MY and QL analyzed the data. MY, JY, and XC wrote the paper.

## Funding

This work was supported by the National Natural Science Foundation of China (81400283 and 81470502).

### Conflict of interest statement

The authors declare that the research was conducted in the absence of any commercial or financial relationships that could be construed as a potential conflict of interest.
